# The Role of *Cutibacterium acnes* in Intervertebral Disc Inflammation

**DOI:** 10.3390/biomedicines8070186

**Published:** 2020-06-30

**Authors:** Bettina Schmid, Oliver Hausmann, Wolfgang Hitzl, Yvonne Achermann, Karin Wuertz-Kozak

**Affiliations:** 1 Department of Dermatology, University Hospital Zurich, Gloriastrasse 31, 8091 Zurich, Switzerland; bettina.schmid@usz.ch; 2 Faculty of Science, University of Zurich, Gloriastrasse 31, 8091 Zurich, Switzerland; 3 Neuro- and Spine Centre, Klinik St. Anna, St. Anna Street 32, 6006 Lucerne, Switzerland; ohausmann@hin.ch; 4 Department of Neurosurgery, Inselspital Berne, University of Berne, Murtenstrasse 11, 3010 Berne, Switzerland; 5 CABMM Center for Applied Biotechnology and Molecular Medicine, University of Zurich, 8057 Zurich, Switzerland; yvonne.achermann@usz.ch; 6 Research Office (Biostatistics), Paracelsus Medical University, Strubergasse 20, 5020 Salzburg, Austria; Wolfgang.Hitzl@pmu.ac.at; 7 Research Program Experimental Ophthalmology and Glaucoma Research, Paracelsus Medical University, Müllner Hauptstr. 48, 5020 Salzburg, Austria; 8 Department of Ophthalmology and Optometry, Paracelsus Medical University Salzburg, 2020 Salzburg, Austria; 9 Division of Infectious Diseases and Hospital Epidemiology, University Hospital Zurich, Rämistrasse 100, 8091 Zurich, Switzerland; 10 Faculty of Medicine, University of Zurich, Rämistrasse 100, 8091 Zurich, Switzerland; 11 Department of Biomedical Engineering, Rochester Institute of Technology (RIT), 106 Lomb Memorial Dr., Rochester, NY 14623, USA; 12 Institute for Biomechanics, D-HEST, ETH Zurich, Hönggerbergring 64, 8093 Zurich, Switzerland; 13 Spine Center, Schön Clinic Munich Harlaching (Academic Teaching Hospital and Spine Research Institute of the Paracelsus Medical University Salzburg), Harlachinger Str. 51, 81547 Munich, Germany

**Keywords:** *Cutibacterium acnes*, Toll-like receptor 2 (TLR2), Toll-like receptor 4 (TLR4), interleukin, low back pain, discogenic back pain, pro-inflammatory cytokines, responders, non-responders

## Abstract

Recently, the role of infection of the intervertebral disc (IVD) with *Cutibacterium acnes* (*C. acnes*) as a contributor to disc-related low back pain (LBP) has been discussed. The aim of this study was to investigate whether and how *C. acnes* contributes to the inflammatory processes during IVD disease. The prevalence of *C. acnes* infection in human IVD tissue was determined by aerobic and anaerobic culture. Thereafter, primary human IVD cells were infected with a reference and a clinical *C. acnes* strain and analyzed for pro-inflammatory markers (gene/protein level). In a subsequent experiment, the involvement of the Toll-like receptor (TLR) pathway was investigated by co-treatment with sparstolonin B, a TLR2/4 inhibitor. We detected *C. acnes* in 10% of IVD biopsies (with either herniation or degeneration). Stimulating IVD cells with both *C. acnes* strains strongly and significantly upregulated expression of Interleukin (IL)-1β, IL-6, IL-8, and inducible nitric oxide synthase (iNOS). IL-6, cyclooxygenase (COX)-2, and iNOS expression was reduced upon TLR2/4 inhibition in 3 out of 5 donors, whereby responders and non-responders could not be differentiated by their basal TLR2 or TLR4 expression levels. We demonstrate that exposure of IVD cells to *C. acnes* induces an inflammatory response that may contribute to the development of discogenic LBP by involving TLR2/4 activation, yet only in a subgroup of patients. Whether the same response will be observed in vivo and where lower inoculums are present remains to be proven in future studies.

## 1. Introduction

Low back pain (LBP) is the primary cause of disability worldwide, with a lifetime prevalence of >80% [1]. The costs associated with LBP are staggering, with, e.g., 2.9 billion CHF of direct costs in Switzerland [2] and >100 billion USD of total costs in the United States [3]. Therefore, LBP represents a massive socio-economic burden. One of the main sources of LBP is the degenerating intervertebral disc (IVD). While disc degeneration is asymptomatic in the majority of people, a subpopulation experiences painful disc degeneration (DD), characterized by degeneration and consequent structural failure of the IVD, in combination with nociception [4]. Another pathological process that is commonly associated with pain development is disc herniation (DH), i.e., structural damage to the outer area of the disc (the annulus fibrosus—AF), enabling the inner nucleus pulposus (NP) to extrude through the damaged fibers. While degeneration of the IVD is a process that is required for DH, it does not necessarily lead to DH, but may be limited to, e.g., microfissures in the endplate or the AF. In both cases, inflammatory processes are known to play a crucial role in pain development. The extruded NP tissue in case of DH has long been known to be inflammatory in nature, with invading immune cells such as macrophages contributing to the inflammatory behavior [5,6,7]. However, IVD cells themselves can also release such pro-inflammatory cytokines [5]. This is specifically relevant in painful DD, where nociception is strongly associated with innervation as well as with the occurrence of chronic inflammation within the tissue, with selected pro-inflammatory cytokines (e.g., IL-6) causing direct nerve irritation [7,8,9]. Several receptors and pathways seem to be implicated in IVD inflammation, such as Toll-like receptors (TLRs), which can be activated by so-called damage-associated molecular patterns (DAMPs)) as well as the NF-kB and MAPK pathways [10,11,12,13].

More recently, studies on the role of bacterial infection as a contributor to disc-related LBP have gained intensive interest. As early as in 2001, Stirling et al. reported that 53% of IVD tissues derived from micro-discectomy were characterized as infected with gram-positive microorganisms, with *Cutibacterium C. acnes* (formerly *Propionibacterium acnes*) being the dominant bacteria [14]. The ability of *C. acnes* to infiltrate IVD tissue in case of DH has been delineated extensively and most studies thus far have been conducted on DH samples/patients [15]. However, microfissures often preceding DH can already allow for *C. acnes* infiltration (especially as microfissures also support neovascularization/ angiogenesis) and thus propagate an immunologic response during DD [7].

However, as *C. acnes* is one of the most abundant bacteria on human skin [16], it is also a proven contaminant of human clinical samples, e.g., through contact with the patient’s skin or laboratory reagents. In fact, a recent study investigated the presence of *C. acnes* DNA in 20 different sample types using existing next-generation sequencing data and found it to be present in the majority of samples, albeit at low concentrations [17]. Consequently, the detection of *C. acnes* in the IVD led to an intensive debate on the topic “contamination versus low grade infection”, which was further incited by Albert et al. in 2008, publishing promising results of antibiotic treatment for patients with LBP (with Modic changes Type 1, prospective uncontrolled trial, *n* = 32) [18]. This study was later followed-up by a double-blind randomized clinical controlled trial (LBP patients with Modic changes Type 1, *n* = 162), demonstrating superiority of antibiotic treatment over placebo, with improvements in lumbar pain and leg pain [19]. Underlining the debate, Braten et al. did not find a beneficial effect of antibiotics in their study [20]. Nonetheless, as bacteria can produce pathogen-associated molecular patterns (PAMPs), which in turn can function as TLR activators [21], the association between *C. acnes* infection in the IVD and the occurrence of discogenic LBP may be related to the induction of inflammatory processes within the IVD tissue.

Therefore, the aim of this study was to investigate whether and how *C. acnes* contributes to the inflammatory processes during IVD diseases (DD and DH), with a focus on the potential involvement of the TLR2/4 pathway.

## 2. Results

### 2.1. Prevalence of C. acnes Infection in 30 IVD Biopsies

We assessed 30 IVD biopsies for bacterial growth. The biopsies originated from patients operated due to DH or DD, and their characteristics are summarized in Table 1. Seven out of 30 biopsies (23.3%) were culture positive for any bacteria (see Table 1), but only three (10%) were positive for *C. acnes* after a prolonged cultivation time (in broth only at day 12). All three cultures became positive after a prolonged cultivation time using enrichment broth. The biopsies with detection of *C. acnes* originated from two patients with DH and from one with DD. *C. acnes* infection could not be correlated with any patient/tissue characteristics.

### 2.2. Changes in IVD Cell Gene Expression upon Exposure to C. acnes

To assess the impact of *C. acnes* infection on IVD cells, we analyzed the gene expression of different inflammation markers.

Cell viability, measured as metabolic activity (MTT assay), was higher than 80% in all three *C. acnes* concentrations measured (multiplicities of infection (MOI) 1, 10, and 100). MOI 100 was chosen in subsequent experiments to achieve the clearest results (the metabolic activity of MOI 100 is shown in Supplementary Figure S1).

**Figure 1:**
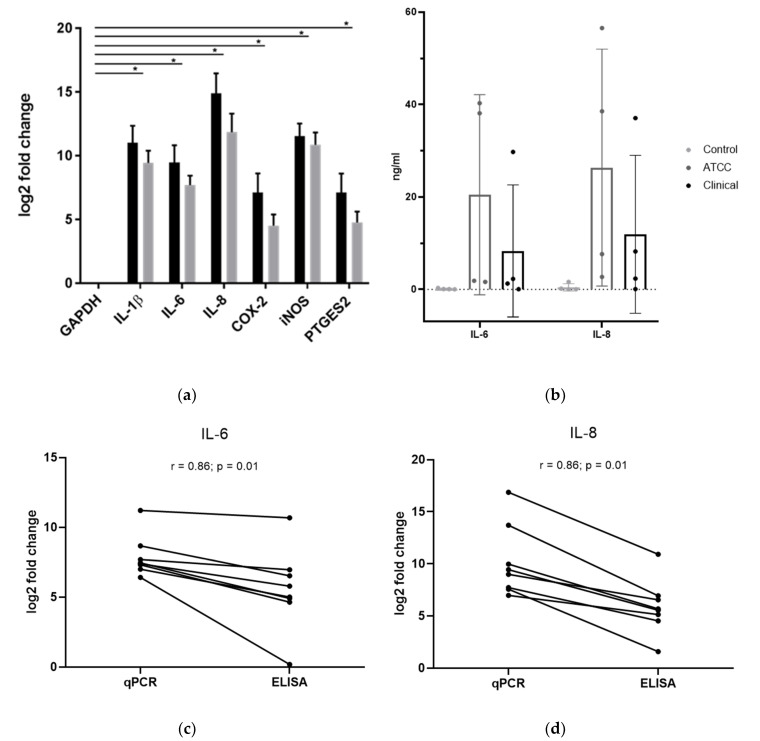
*C. acnes*-induced changes of pro-inflammatory markers in intervertebral disc (IVD) cells. The black bars display results obtained with the ATCC *C. acnes* strain, whereas light grey shows results obtained with the clinical strain (*n* = 4 donors with 2 technical replicates for each donor). (**a**) Gene expression of Interleukin (IL)-1β, IL-6, IL-8, cyclooxygenase (COX)-2, inducible nitric oxide synthase (iNOS), and prostaglandin E synthase 2 (PTGES2). Fold changes (2^−ΔΔ*C*t^) are indicated on a logarithmic scale and normalized to the endogenous control glyceraldehyde 3-phosphate dehydrogenase (GAPDH) as well as the negative control; * *p* < 0.05. Data represent the mean with standard deviation (SD). (**b**) Protein expression of IL-6 and IL-8 in ng/mL. Mean with SD is shown. (**c**) Correlation of IL-6 between qPCR and ELISA results. (**d**) Correlation of IL-8 between qPCR and ELISA results. Spearman correlation coefficients: r = 0.86; *p* = 0.01.

We examined the gene expression of IL-1β, IL-6, IL-8, cyclooxygenase 2 (COX-2), inducible nitric oxide synthase (iNOS), and human prostaglandin E synthase 2 (PTGES2) in human IVD cells (mixture of NP and AF cells with different NP/AF ratios depending on the biopsy) infected with *C. acnes* to assess the extent of inflammation. Figure 1a shows the differences in gene expression of untreated cells and infected cells (either with the ATCC *C. acnes* strain (black) or the clinical *C. acnes* strain (light grey)), displayed as logarithmic fold change. Gene expression of all measured markers was highly and significantly upregulated (all *p* < 0.034), with the highest increases for IL-8 and iNOS, followed by IL-1β and IL-6. Mean fold changes were 2069 (ATCC strain (A)) and 695 (clinical strain (C)) for IL-1β, 714 (A) and 208 (C) for IL-6, 30276 (A) and 3677 (C) for IL-8, 140 (A) and 23 (C) for COX-2, 2969 (A) and 1864 (C) for iNOS, and 139 (A) and 27 (C) for PTGES2. Fold changes varied greatly between individual donors. Stimulation with C. acnes furthermore induced a moderate upregulation of TLR2 and a minor downregulation of TLR4 (data not shown). Since the ATCC and the clinical strains had distinct morphologies regarding hemolytic activity and presumably differences in virulence, we were interested in the comparison of the gene expression levels of both strains. However, no statistical significance was detected for any of the genes.

### 2.3. Changes in IVD Cell Protein Expression upon Exposure to C. acnes

To confirm the elevated levels of pro-inflammatory cytokines, which were discussed in Section 2.2, we performed experiments on the protein level by measuring protein concentrations with ELISA. In this experiment, IL-1β, IL-6, and IL-8 levels were measured. IL-1β levels were below the detection limit in the control as well as the infected cells, but also had higher Ct values than IL-6 and IL-8 in qPCR experiments. Figure 1b shows the protein expression levels of IL-6 and IL-8. The IL-6 levels were 0.1 ± 0.1 ng/mL in the untreated control, 20.5 ± 18.7 ng/mL in the cells infected with the ATCC strain, and 8.3 ± 12.4 ng/mL in the cells infected with the clinical strain. Accordingly, the IL-8 levels were 0.5 ± 0.7 ng/mL in the untreated control, 26.3 ± 22.2 ng/mL in the cells infected with the ATCC strain, and 11.9 ± 14.8 ng/mL in the cells infected with the clinical strain. Although both cytokines showed highly increased concentrations in infected cells, these results did not reach statistical significance due to high fluctuations between individual donors (IL-6 ATCC: *p* = 0.078; IL-6 clinical: *p* = 0.17; IL-8 ATCC: *p* = 0.065; IL-8 clinical: *p* = 0.127).

The results on gene and protein level correlate by looking at the different donors as shown in Figure 1c (IL-8) and Figure 1d (IL-6) with a Spearman correlation coefficient of 0.8571 (*p* = 0.011). qPCR and ELISA results showed a high correlation, meaning that donors with high fold changes also showed high concentrations in ELISA experiments.

### 2.4. Involvement of TLR2/4

Sparstolonin B is a potent TLR2/4 inhibitor, and according to our hypothesis of TLR2/4 involvement in *C. acnes*-induced inflammation, cytokine levels should decrease after stimulation with it.

Inhibition of marker genes varied greatly between the different donors. Donors in which the expression of the different target genes was partially inhibited are defined as responders (*n* = 3), whereas donors in which sparstolonin B showed no inhibitory effect are described as non-responders (*n* = 2).

**Figure 2:**
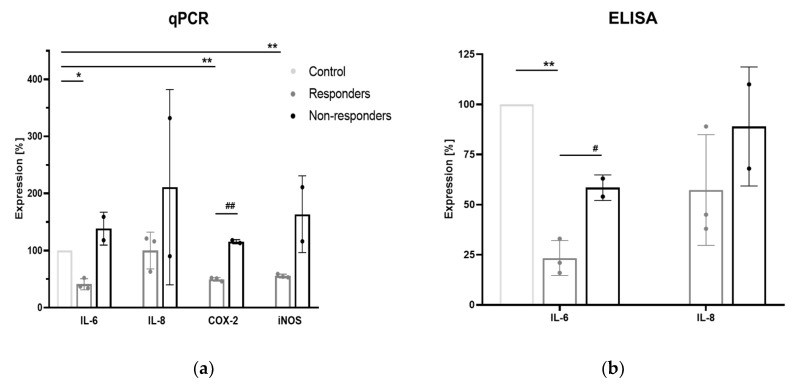
Expression of IL-6, IL-8, COX-2, and iNOS after Toll-like receptor (TLR)2/4 inhibition in IVD cells. The light grey columns display the control (IVD cells with *C. acnes* infection but without sparstolonin B treatment), whereas responders are colored in grey and non-responders in black (responders: *n* = 3, non-responders: *n* = 2). (**a**) Gene expression levels of qPCR results (**b**) Protein expression levels obtained with ELISA. * *p* < 0.05 relative to control, ** *p* < 0.01 relative to control, # *p* < 0.05 between responders and non-responders, ## *p* < 0.01 between responders and non-responders. Data are shown in mean with SD.

Figure 2a shows gene expression of IL-6, IL-8, COX-2, and iNOS after TLR2/4 inhibition in responders (grey) and non-responders (black) compared to the control without TLR2/4 inhibition. Mean expression of the responders was 41 ± 7.9% for IL-6, 100 ± 26.2% for IL-8, 49.7 ± 2.6% for COX-2, and 55.3 ± 2.6% for iNOS. Mean expression of the non-responders was 138.5 ± 20.5% for IL-6, 211 ± 121% for IL-8, 115.5 ± 2.5% for COX-2, and 163.5 ± 47.5% for iNOS. IL-6, COX-2, and iNOS expressions were significantly decreased after inhibition of TLR2/4 in the responder cells (IL-6: *p* = 0.009; COX-2: *p* = 0.001; iNOS: *p* = 0.002), whereas there was no observed inhibition in all the investigated genes in the non-responders as well as of IL-8 expression in the responders. Furthermore, we observed a significant difference within responders and non-responders in the expression of COX-2, with a *p*-value of *p* = 0.0002.

Figure 2b displays protein expression levels obtained with ELISA. Expression levels were decreased more extensively compared to the qPCR results, with significant reductions of IL-6 in the responders (*p* = 0.004) and a significant difference between responders and non-responders (*p* = 0.017). Mean expression levels were 23.3 ± 7.1% (responders) and 58.5 ± 4.5% (non-responders) for IL-6. For IL-8, no significances were reached and mean responses were 57.3 ± 22.6% (responders) and 89.0 ± 21.0% (non-responders).

### 2.5. Basal Expression of TLR2/4

**Figure 3:**
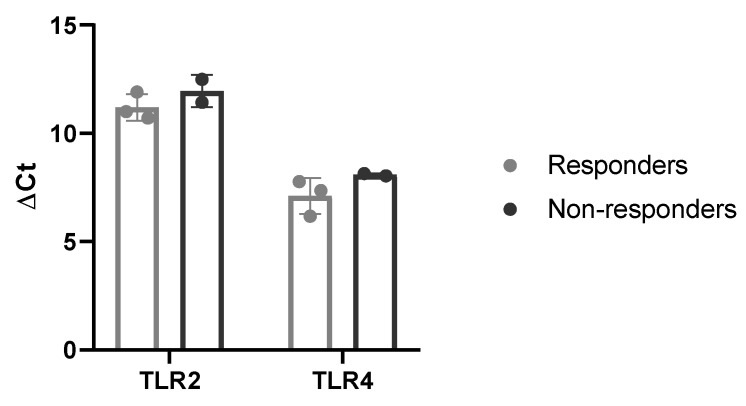
Basal expression levels of TLR2 and TLR4 in responders (grey, *n* = 3) and non-responders (black, *n* = 2). Data represents mean with SD.

Since some donors responded to the TLR2/4 inhibition whereas others did not, we were interested in basal expressions of TLR2 and TLR4. In Figure 3, ΔCt values of the untreated cells were compared between responders and non-responders. However, responders and non-responders showed no significant differences in the TRL2 and TLR4 basal expression.

## 3. Discussion

The role of *C. acnes* infection in the development of back pain has been an extensive matter of debate for a while, following publications reporting on a potential benefit of antibiotics treatment in back pain patients [18,19] as well as studies describing a high prevalence (46%) of *C. acnes* infection in herniated IVD tissue [22]. In this study, we only found *C. acnes* in 10% of human IVD biopsies when culturing the pathogen in broth only and never on direct agar plating, pointing towards a very low inoculum in IVD tissue. Interestingly, *C. acnes* positivity was not only found in DH samples (as previously reported [19]), but also in a DD sample, indicating that degenerative processes with associated development of, e.g., microfissures can serve as an entry point for bacteria. Due to the limited sample number, we could not determine whether any specific patient or tissue characteristics predispose for *C. acnes* infection. Although care was taken during surgical preparation and biopsy excision, the potential risk of sample contamination in the pre-analytical phase cannot be ruled out, which would result in a slightly lower prevalence of *C. acnes* growth than the stated 10%. Our findings are comparable to the 15.6% recently reported by Ahmed-Yahia [23]. Using molecular testing following enrichment cultures of 14 days, Yuan et al. [24] recently described a *C. acnes* prevalence of 20% (15 out of 76). However, the risk for false positive results may be increased using molecular methods (which has been commonly used in past IVD research [25]) compared to positive conventional cultures with subsequent identification of growing colonies, thus indicating a possible overestimation of the role of *C. acnes* in IVD pathologies and back pain.

It has recently been hypothesized that *C. acnes* infection of the IVD might lead to back pain due to the induction of inflammatory tissue responses. In support of this theory, we were able to show strong upregulation of gene expression of IL-1β, IL-6, IL-8, and iNOS following exposure of IVD cells (isolated from DH and DD biopsies) to a clinical and a laboratory *C. acnes* strain. This increase could be confirmed on the protein level in IL-6 and IL-8, with a good correlation to gene expression. Dudli et al. also demonstrated an increase in IL-1, IL-6, and IL-8 mRNA expression in IVD cells upon co-culture (MOI 10 and MOI 100), however, at a much lower level (typically < 10 fold); only in 6 out of 10 donors and without any analysis of the protein level [26]. However, it has to be pointed out that in vitro studies can only serve as a model system that does not fully represent the human in vivo situation. In fact, Yuan et al. were able to verify the increased expression of IL-8 in *C. acnes* positive IVD biopsies, whereas IL-1β and IL-6 were not significantly different from uninfected controls in this explant study [24]. Despite being a tissue with an extremely low cellularity [27], in vivo ratios of IVD cells to *C. acnes* are likely lower than in vitro MOIs. We chose MOI 100 to induce a clear, model-like response in which the involvement of the TLR pathway could be examined clearly. Therefore, inflammatory IVD cell responses are probably lower in affected patients than observed in vitro.

A potential bias in the extent of inflammation could be introduced due to the composition of disc cells (mix of NP and AF or predominant fraction of one type) or invading inflammatory cells (particularly macrophages) [28]. Despite much research activity, no markers have yet been established by the research community that conclusively distinguish NP cells from AF cells, although novel possible candidates are emerging [29]. During DD, the NP tissue undergoes a loss in proteoglycans with a concomitant shift of Coll-2 to Coll-1, which increases its similarity to AF tissue, making zonal differentiation challenging [30]. The potentially invading tissue macrophages upon structural failure of the IVD are well known to have very limited proliferation capacity and their self-renewal capacity is determined by specific environmental conditions not present in the IVD cell expansion setup used in this study [31]. However, any remaining tissue macrophages contained in the culture would be fairly reflective of the in vivo situation.

Following confirmation of the induction of an inflammatory response, we were interested in identifying the underlying molecular mechanism. We previously showed the relevance of the TLR pathway in IVD pathologies [32], specifically in IVD inflammation [12,33]. Furthermore, *C. acnes* has been shown to activate TLR2 in numerous cell types, such as monocytes [34], human embryonic kidney (HEK) 293 cells [34], and keratinocytes [35], typically leading to subsequent activation of the NF-kB pathway. For an extensive summary of the interplay between *C. acnes* and TLR2 activation in the context of acne vulgaris, we refer the reader to a recent review article by Zhang et al. [21]. In IVD cells, *C. acnes* was shown to induce apoptosis via TLR2 activation and subsequent induction of the c-Jun N-terminal kinase (JNK) pathway [36]. Our own data also demonstrated involvement of the TLR2/4 pathway in IVD cell inflammation following exposure to *C. acnes*, but only in a subgroup of patients (so-called responders). Interestingly, responders did not possess a higher basal mRNA expression of TLR2 or TLR4 or dissimilar changes in their expression upon *C. acnes* stimulation than non-responders, suggesting that other factors or mechanisms must contribute to this disparate response pattern amongst different donors. However, due to a relatively small sample size, these questions could not be answered in the present study but will be investigated in future projects focusing on genetic mutations. An interesting candidate in that context will be analysis of the role of *nucleophosmin* gene mutations, which was earlier shown to distinguish TLR responders and non-responders in human acute myeloid leukemia cells [37].

In summary, the exposure of IVD cells to *C. acnes* induced an inflammatory response with elevated IL-6 and IL-8 expression levels in vitro, which (for IL-6) were mediated by TLR2/4 activation in a subgroup of patients. While most *C. acnes* studies focus on DH biopsies/patients, our cell culture studies showed that *C. acnes* induced in inflammatory response in cells stemming from both, DD, and DH. In vivo, microfissures and associated angiogenesis may be sufficient to allow for *C. acnes* infiltration [7]. Due to the very low inoculum in IVD tissue, the response rate in vivo is, however, likely reduced. Nonetheless, this study provided a theoretical molecular link between *C. acnes* infection and discogenic back pain through induction of chronic inflammation that can build the basis for further investigations.

## 4. Materials and Methods

### 4.1. Detection of C. acnes in Clinical Samples of IVD Biopsies

Degenerated IVDs were removed by posterior discectomy of patients with DD or DH. To avoid tissue biopsy contamination during surgery, the following prevention strategies were routinely performed: (1) skin preparation with povidone iodine/alcohol three times, (2) anti-microbial prophylaxis with cefuroxime 30 min before skin incision, and (3) tissue removal using unused surgical instruments and under meticulous avoidance of touching the skin with neither the tissue nor the instruments. As the prevalence of *C. acnes* in IVDs has been described to be 13–44% [15], we aimed at a sample number of at least *n* = 25.

Biopsies were immediately sent to the research bacterial laboratory of the University Hospital Zurich where the biopsies were cut into small pieces and homogenized (Polytron PT1200 homogenizer, Kinematica, Lucerne, Switzerland) for 1 min. On Brain Heart Infusion (BHI) agar (70138, Sigma, St. Louis, MO, USA), 100 μL of the homogenized liquid were plated for aerobic (three days) and anaerobic culture (10 days). In addition, the whole biopsy was inoculated in thioglycolate broth (221196, Becton Dickinson, Franklin Lakes, NJ, USA) to enrich the culture. Thioglycolate medium was inspected every third day for cloudiness. Subsequently, 100 μL of cloudy liquid were plated onto aerobic and anaerobic agar plates for microbial growth and further identification. If the thioglycolate broth culture was negative for 10 days, a final blind subculture on brucella agar (10% sheep blood agar (Becton Dickinson, Franklin Lakes, NJ, USA) with hemin and vitamin K1 (Sigma, St. Louis, MO, USA)) was done for another two to three days. All growing colonies were identified by MALDI-TOF (matrix-assisted laser desorption ionization–time of flight) using a Bruker MALDI Biotyper (Becton Dickinson, Franklin Lakes, NJ, USA).

### 4.2. Isolation of IVD Cells and Primary Cell Culture

Mixed IVD tissue was collected from 9 patients undergoing spinal surgery due to DD and DH (further information is shown in Table 2). Infection experiments where conducted on tissue from 4 patients, inhibition experiments on tissue from 5 patients. All patients granted written informed consent and the study was approved by the Ethic Committee Zurich (#EK-16/05 (01/2009) and #2019-00736 (05/2019)). The tissue was cut into pieces for enzymatic digestion with 0.2% collagenase NB4 (17454, Serva, Heidelberg, Germany) and 0.3% dispase II (04942078001, Roche, Basel, Switzerland) for 12–18 h at 37 °C. After filtration with a 70 µM cell strainer, the cells were suspended in Dulbecco’s Modified Eagle’s Medium (DMEM/F12, D8437, Sigma, St. Louis, MO, USA) supplemented with 10% fetal calf serum (FCS, F7524, Sigma, St. Louis, MO, USA) and 1% Antibiotics-Antimycotics (A/A, 15240062, Gibco, Carlsbad, CA, USA). Sub-cultures up to three passages were used for further experiments. The IVD cultures contained a mixture of NP and AF cells, with some biopsies being predominantly NP, some being predominantly AF and other likely being an even mixture. Cells were maintained at 37 °C with 5% CO_2_, and media were changed twice a week.

### 4.3. Bacterial Culture

To observe the impact of their origin, two *C. acnes* strains were used for the experiments: the reference strain ATCC 11,827 used for biofilm studies and a clinical strain isolated from a patient with a spine infection (personal clinical strain collection Y69) [38,39]. The strains were cultured on Brucella agar (10% sheep blood agar (Becton Dickinson, Franklin Lakes, NJ, USA) with hemin and vitamin K1 (Sigma, St. Louis, MO, USA)) for 72 h under anaerobic conditions at 37 °C. For sub-cultivations and liquid cultures, BHI agar (70138, Sigma, St. Louis, MO, USA) and broth (211059, BD, Franklin Lakes, NJ, USA) were used.

### 4.4. Metabolic Activity Assay (MTT)

To assess the influence of co-culturing IVD cells and *C. acnes* and of treatment with sparstolonin B (SML1767, Sigma, St. Louis, MO, USA) on metabolic activity, the MTT assay (3-[4–dimethylthiazol-2-yl]μ-2,5-diphenyl tetrazolium bromide formazan, M2003, Merck, Darmstadt, Germany) was used. IVD cells were seeded and co-cultured with *C. acnes* (MOI 1, 10, and 100) with or without stimulation of different sparstolonin B concentrations (10, 15, 20, 25, 50, and 100 μM). Extracellular bacteria were killed by incubation for 2 h with penicillin G (0.128 mg/mL, 13752, Merck, Darmstadt, Germany). The cells were then incubated with MTT in DMEM/F12 (5 mg/mL) for 2 h at 37 °C, 0.04 M HCl (H1758, Sigma, St. Louis, MO, USA) in isopropanol (2-propanol, I9516, Sigma, St. Louis, MO, USA) was used as a solubilization solution, and the absorbance was measured at 570 nm. Metabolic activity was calculated relative to the untreated control.

### 4.5. Infection of IVD Cells with C. acnes

5 × 10^5^ IVD cells in 3 mL media (approximately 1.7 × 10^5^ cells/mL) were seeded onto 6-well plates and incubated for 24 h in DMEM/F12 supplied with 10% FCS but without A/A to allow co-cultivation with *C. acnes*. The bacteria were grown in liquid culture for 48 h before they were added to the IVD cells in a 100:1 multiplicity of infection (MOI).

To guarantee an immediate contact between the cells and the bacteria, the plates were centrifuged at 250× *g* for 5 min. The cultures were harvested 24 h post infection. The supernatants were sterile filtered and frozen at −80 °C for further ELISA experiments, whereas the pellets were washed three with Phosphate Buffered Saline (PBS, pH 7.4, 10010-015, Gibco, Carlsbad, CA, USA) to get rid of all the bacteria. The IVD cells were lysed with Qiagen RNeasy RLT buffer (74106, Qiagen, Hilden, Germany).

### 4.6. Infection of IVD Cells with C. acnes with TLR2/4 Inhibition

For the inhibition assay, the experiment explained in Section 2.4 was repeated with one difference. The IVD cells were pre-treated with 25 µM sparstolonin B 1 h prior to infection with bacteria to inhibit TLR2/4.

### 4.7. RNA Isolation and Gene Expression Analysis

RNA was isolated with the Qiagen RNeasy Mini Kit (74106, Qiagen, Hilden, Germany) according to the manufacturer’s instructions. RNA concentration and purity were measured with the NanoDrop (ND-1000, Thermo Fisher Scientific, Waltham, MA, USA). In a total volume of 60 µL, 0.85–1.7 µg RNA (depending on concentration) were transcribed to cDNA using the reverse transcription kit (4374966, Applied Biosystems, Foster City, CA, USA). Gene expression of the target genes shown in Table 3 was analyzed with qPCR (CFX96 Touch™ Detection System, Biorad, Hercules, CA, USA), using 30 ng of cDNA/reaction. The Ct values were normalized to the endogenous control glyceraldehyde 3-phosphate dehydrogenase (GAPDH) and to the untreated cells and displayed as fold change (2^−ΔΔ*C*t^).

### 4.8. ELISA

The supernatants, which were collected from the infection and inhibition experiments, were further used for the protein analysis with ELISA. Levels of Interleukin-6 and IL-8 were tested with the BD OptEIA human ELISA kits (IL-6 555220, IL-8 555244, BD Biosciences, San Jose, CA, USA) according to the manufacturer’s instructions. Based on the standard curves, IL-6/-8 concentrations were calculated.

Human IL-1β was measured using a multiplexed particle-based flow cytometric cytokine assay [40]. The IL-1β kit was purchased from Biotechne (Oxon, UK). The procedure closely followed the manufacturer’s instructions. The analysis was conducted using a conventional flow cytometer (Guava EasyCyte Plus, Millipore, Zug, Switzerland).

### 4.9. Statistical Analysis

Data consistency was checked, and data were screened for outliers. Continuous variables were tested for normality by using Kolmogorov–Smirnov tests. One-sample Wilcoxon Signed Rank tests (one sided) were used to test whether medians were larger 1. Corresponding paired tests were tested two sided. All reported tests were two-sided, and *p*-values < 0.05 were considered statistically significant. All statistical analyses in this report were performed by use of PASW 21 (IBM SPSS Statistics for Windows, Version 21.0., Armonk, NY), NCSS 10 Statistical Software (2015), NCSS, LLC. Kaysville, UT, USA and STATISTICA 13 (Hill, T., and Lewicki, P. Statistics: Methods and Applications. StatSoft, Tulsa, OK, USA).

## Figures and Tables

**Table 1:** Characterization of 30 patients undergoing spinal surgery.

Characterization of Patients	*n* (%)
Age, median (range)	55 (20–78)
Sex, female	12 (40)
Smoker	10 (33)
Previous cortisone infiltration	8 (26.7)
**Characterization of Biopsies**	
*Spinal Area*	
Cervical	11 (36.7)
Lumbar	19 (63.6)
*Degree of disc degeneration–Pfirrmann Score ^1^*	
II	3 (10)
III	17 (56.7)
IV	9 (30)
V	1 (3.3)
*Modic changes ^2^*	
I	12 (40)
II	15 (50)
Unknown	3 (10)
*Tissue*	
Annulus fibrosus	6 (20)
Nucleus pulposus	19 (63.3)
Mix	5 (16.7)
*Disease*	
Disc herniation	10 (33.3)
Disc degeneration	20 (66.7)
*Positive Culture Result*	7 (23.3)
*Cutibacterium acnes*	3 (10)
Coagulase-negative staphylococci ^3^	3 (10)
Unknown ^4^	1 (3.3)

^1^ Pfirrmann Score = a grading system for disc degeneration on MRI T2-spin-echo weighted images. ^2^ Modic changes = a grading system for pathological changes in the bones of the spine (body of the vertebrae and in the end plate of the neighboring disc). ^3^ *Staphylococcus hominis* (*n* = 1), *Staphylococcus saprophyticus* (*n* = 1), *Staphylococcus capitis/Staphylococcus epidermidis* (*n* = 1). ^4^ Unknown pathogen (unsuccessful identification by MALDI-TOF, DNA not stored for molecular analysis).

**Table 2:** Characterization of 9 patients providing biopsies for IVD cultures.

Characterization of Patients	*n* (%)
Age, median (range)	60 (25–74)
Sex, female	4 (44.5)
**Characterization of Biopsies**	
*Spinal Area*	
Cervical	1 (11.1)
Lumbar	8 (88.9)
*Degree of disc degeneration–Pfirrmann Score ^1^*	
II	2 (22.2)
III	3 (33.3)
IV	4 (44.5)
*Modic changes ^2^*	
0	2 (22.2)
I	2 (22.2)
II	4 (44.5)
Unknown	1 (11.1)
*Diagnosis*	
Disc herniation	5 (55.6)
Disc degeneration ^3^	4 (44.4)

^1^ Pfirrmann Score = a grading system for disc degeneration on MRI T2-spin-echo weighted images. ^2^ Modic changes = a grading system for pathological changes in the bones of the spine (body of the vertebrae and in the end plate of the neighboring disc). ^3^ Some cases had a concomitant pathology, such as listhesis.

**Table 3:** TaqMan primers used for qPCR.

Target Gene	Assay Identification Number
COX-2	Hs00153133_m1
GAPDH	Hs02758991_g1
IL-1β	Hs00174097_m1
IL-6	Hs00174131_m1
IL-8	Hs00174103_m1
iNOS	Hs01075521_m1
PTGES2	Hs00228159_m1
TLR2	Hs00152932_m1
TLR4	Hs00152939_m1

Abbreviations: COX-2, cyclooxygenase-2; GAPDH, glyceraldehyde 3-phosphate dehydrogenase; IL, interleukin; iNOS, inducible nitric oxide synthase; PTGES2, human prostaglandin E synthase 2; TLR, toll-like receptor.

## Supplementary Materials

Supplementary materials can be found at https://www.mdpi.com/2227-9059/8/7/186/s1.

## Abbreviations

IVD	Intervertebral disc
*C. acnes*	*Cutibacterium acnes*
LBP	Low back pain
TLR	Toll-like receptor
IL	Interleukin
iNOS	Inducible nitric oxide synthase
COX	cyclooxygenase
DD	Disc degeneration
DH	Disc herniation
AF	Annulus fibrosus
NP	Nucleus pulposus
DAMP	Damage-associated molecular pattern
PAMP	Pathogen-associated molecular pattern
MOI	Multiplicity of infection
PTGES2	Human prostaglandin E synthase 2
JNK	c-Jun N-terminal kinase
BHI	Brain Heart Infusion
MALDI-TOF	Matrix-assisted laser desorption ionization–time of flight
DMEM	Dulbecco’s Modified Eagle’s Medium
FCS	Fetal calf serum
A/A	Antibiotics/antimycotics
MTT	3-[4–dimethylthiazol-2-yl]-2,5-diphenyl tetrazolium bromide formazan
PBS	Phosphate Buffered Saline
