# Supplementary material for: The Role of Cutibacterium acnes in Intervertebral Disc Inflammation
Source: Biomedicines. 2020 Jun 30;8(7):186. doi: 10.3390/biomedicines8070186 (PMC7400222; doi:10.3390/biomedicines8070186)
Supplement: Supplementary file 1 [file biomedicines-08-00186-s001.pdf]

Supplementary materials

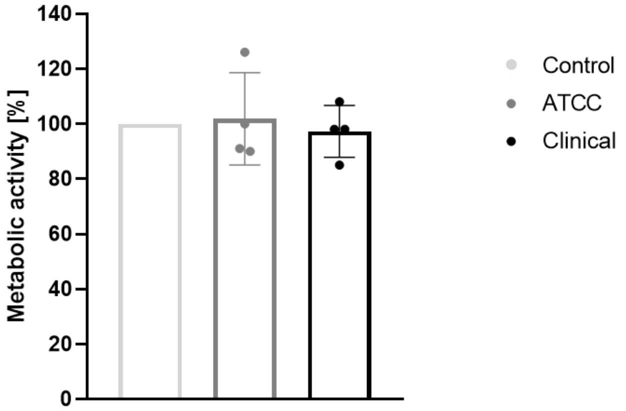

Supplementary Figure 1. Metabolic activity of IVD cells infected with the ATCC (grey) or clinical (black) *C. acnes* strain. Values are normalized to metabolic activity of untreated IVD cells (control in light grey).
